# The Story of the Insane from Year to Year

**Published:** 1895-07-20

**Authors:** 


### THE STORY OF THE INSANE FROM YEAR TO YEAR.

Eighth Series.

### THE CORNWALL ASYLUM, BODMIN.

On January 1st, 1894, there were 714 patients in this asylum, and at the end of the year the number had risen to 725. The admissions during the year were 121; the recoveries, 52; discharged relieved, 2; and deaths, 49. The percentage of recoveries on admissions was 44·8, and that of the deaths was 6·8 on the average number resident. The former is above the average recovery rate in our county asylums, and the latter is below the average death-rate. Twelve of the deaths were due to cerebral and spinal diseases, and 8 to consumption. The chief causes of insanity in the year's admissions, as given in Table X., are: Domestic trouble, 8; religion, 4; heredity, 16; drink, 3; previous attacks, 35; and unknown, 10. The asylum is full (the Commissioners say it is overcrowded), and the medical superintendent thinks fresh cases can only be admitted as vacancies caused by discharge or death occur. In a large county asylum this cannot be regarded as a satisfactory state of things; moreover, the committee and superintendent will probably find that the applications for admissions will exceed the vacancies, and either further crowding must take place or patients must be boarded out in other asylums at a considerably increased cost to the ratepayers. The committee very truly say that the question is one which cannot be "much longer delayed, as however soon the matter is dealt with, it will take five years at least to get the plans for the new buildings sanctioned and the buildings erected." We may remark that they may think themselves lucky if they do it in five years. The committee have formulated a pensions scheme for their officers and servants, and by this scheme anyone at present in their service may retire at the age of 50 and 15 years' service on a pension of one-fortieth of pay and allowances for every year's service. The maximum service to be 26 years. Those officers and servants who enter after January, 1895, will be under the same scheme, except that the minimum age of retiring will be 55. On the whole it may be taken as a fairly liberal scheme; but we do not notice any age stated for compulsory retirement, without which the pension system is shorn of one of its advantages. Then again, in the great majority of cases it will be found that a nurse on the insane waddling about the wards at the age of 55 is not worth her wages. For women, certainly, 50 is old enough for active service in the wards of an asylum. We congratulate Dr. Adams on having steered the scheme to a safe haven, and we firmly believe it will conduce greatly to his obtaining and retaining an efficient staff. In this asylum there is special accommodation for private patients, and these pay from 10s. to £2 2s. a week. The charge to the unions for paupers is 10s. a week. We notice that the farm is credited with a profit of £423 on the year's working, and if this be taken as a criterion the Cornish farmer ought to be a happy man. We shall soon see farmers from other counties migrating to Land's End.

### THE ABERGAVENNY ASYLUM.

This is the asylum for the counties of Monmouth, Brecon, and Radnor. On January 1st, 1894, it contained 935 patients, and on December 31st the numbers had risen to 944. There were 196 admissions, 70 recoveries, 14 discharged relieved, and 75 deaths. The recoveries stand at 35·7 on the admissions, and the deaths at 7·9 on the average number resident. Cerebral and spinal diseases are responsible for 36 of the deaths, enteritis for 1, consumption for 5, and pneunmoia for 5. The latter disease seems to appear more frequently in the death tables of asylums of late years than it formerly did, and we think it oftener assumes an epidemic form in such institutions than it does elsewhere. Intemperance in drink appears as the cause of insanity in 18 of the year's admissions, hereditary influence in 47, previous attacks in 40, and domestic trouble in 6. The asylum is practically full. On the date of the report there was vacant accommodation for only six patients, but if the private patients were discharged the vacancies would rise very considerably. Dr. Glendinning would be wise not to build too much on these possible vacancies, as he would find that many of those private patients, having settlements in one of the three counties, would, if discharged as private cases, be returned as paupers. The asylum is too large to permit of any considerable additions to the main building, but why not build a block for 100 private patients. This plan would solve the difficulty for four or five years in a remunerative manner, and would give the visitors time to decide how and where to erect another asylum. Dr. Glendinning would like to see the length of service of the subordinate staff increased, and he believes this could be done "if the committee could see their way to provide a fixed pension scale, as great uncertainty prevails at present among the attendants as to the amount of pension they are likely to receive." Dr. Glendinning truly adds that the association with the insane has a deteriorating effect mentally and physically, and that it cannot be safely kept up for more than 25 years. We think he is wrong when he says that there are difficulties in the way of fixing a satisfactory scale of pensions. Such a scale has already been drawn up at Cornwall, Northampton, Derby, and at some of the metropolitan asylums. The only difficulty exists in the fact that the Lunacy Act does not permit the granting of more than two-thirds of wages and allowances as a pension. A nurse with maximum wages of £30 cannot receive much pension from her asylum, and it is very desirable that all the nurses and all the unmarried attendants should join the National Pension Fund for Nurses. A nurse will endeavour to save her premium for this fund. Money is thus saved which otherwise would have been wasted, and when the day of retiring comes it would be found that the two pensions joined afford a comfortable income. The visitors say that "under the able management of the Medical Superintendent, the efficiency and comfort of the establishment have been fully maintained."

### THE MIDDLESEX COUNTY ASYLUM AT TOOTING.

There were 1,116 patients in residence on January 1st, 1894, and 1,121 on December 31st. The admissions reached 357, the recoveries 157, discharged relieved 29, and the deaths 98. The recoveries were 47·1 per cent. on the admissions, and the deaths 8·7 on the average number of resident. Forty-one of the deaths were caused by cerebral diseases; only 7 died of consumption, but we notice that 9 died of pneumonia. Intemperance in drink is the stated cause of insanity in 36 of the admissions, hereditary tendency in 56, previous attacks in 70, and in 73 the cause was unknown. The asylum is rather more than full, and as it is stated in Dr. Hill's report that there is an annual increase of 52 patients it is clear that steps should be taken to meet this increase. To enlarge an asylum already containing over 1,100 patients would be a grievous mistake, and it is to be hoped the Committee of Visitors will realise this and erect a new asylum for the county. The report of the Visitors is a very long one, and deals with several matters usually found in the reports of medical superintendents. The Visitors say that "Dr. Gardiner Hill's able superintendence has again contributed largely to the success of the year's work." The cost of maintenance is 10s. 8¾d. per head per week.

